# Bone Targeting Agents in Patients with Prostate Cancer: General Toxicities and Osteonecrosis of the Jaw

**DOI:** 10.3390/curroncol29030142

**Published:** 2022-03-05

**Authors:** Veronica Mollica, Giacomo Nuvola, Elisa Tassinari, Maria Concetta Nigro, Andrea Marchetti, Matteo Rosellini, Alessandro Rizzo, Costantino Errani, Francesco Massari

**Affiliations:** 1 Medical Oncology, IRCCS Azienda Ospedaliero-Universitaria di Bologna, 40138 Bologna, Italy; giacomo.nuvola@studio.unibo.it (G.N.); elisa.tassinari@studio.unibo.it (E.T.); mariaconcetta.nigro@studio.unibo.it (M.C.N.); andrea.marchetti12@studio.unibo.it (A.M.); matteo.rosellini@studio.unibo.it (M.R.); francesco.massari@aosp.bo.it (F.M.); 2 Struttura Semplice Dipartimentale di Oncologia Medica per la Presa in Carico Globale del Paziente Oncologico ‘Don Tonino Bello’, IRCCS Istituto Tumori ‘Giovanni Paolo II’, Viale Orazio Flacco 65, 70124 Bari, Italy; a.rizzo@oncologico.bari.it; 3 Department of Orthopaedic Oncology, IRCCS Istituto Ortopedico Rizzoli, Via Pupilli 1, 40136 Bologna, Italy; costantino.errani@ior.it

**Keywords:** bone metastasis, bone targeting agents, CRPC, denosumab, HSPC, osteonecrosis of the jaw, prostate cancer, zoledronic acid

## Abstract

Introduction: Bone metastases are the most frequent site of secondary localization of prostate cancer (PCa) and are present in about 90% of cases of advanced disease. Consequently, an adequate management of bone involvement is of pivotal importance in the therapeutic approach and skeletal-related events (SREs) need to be closely monitored and promptly assessed and treated. Bone targeting agents (BTAs), consisting in bisphosphonates and denosumab, are an essential part of the treatment of metastatic prostate cancer that accompanies systemic treatments throughout the most part of the history of the disease. Activity and safety of bone targeting agents: These treatments are correlated to better outcomes in terms of reduction of SREs and, in metastatic castration resistant setting, of increased overall survival (OS), but several important adverse events have to be managed and prevented. Of these, osteonecrosis of the jaw (ONJ) is extremely invalidating and should be managed with a special attention. Discussion: The role of BTAs in prostate cancer is pivotal throughout many stages of the disease, but several toxicities should be quickly recognized and treated. We aim at recollecting evidence on clinical benefit of BTAs, common and specific toxicities, and explore the pathophysiology and clinical aspects of osteonecrosis of the jaw. We present a review of the literature to report the role of the different types of bone targeting agents in the management of prostate cancer with bone metastases with a particular focus on common toxicities and ONJ to recollect current evidences on the activity of these compounds and the correct management of their adverse events.

## 1. Introduction

Bone health should be a pivotal clinical aspect to address in the management of PCa during the whole natural history of the disease, considering that about 90% of patients present skeletal involvement [1]. The need to properly address the bone health issue in PCa patients is due to the extremely high bone fragility correlated to several aspects: the pre-existing bone damage induced by ageing and comorbidities, the side effects of androgen deprivation therapy (ADT)–current standard of care in the management of both hormone-sensitive (HSPC) and castration resistant prostate cancer (CRPC)–and the high PCa bone tropism, intrinsic characteristic of this disease [2]. Different anatomical and molecular patterns can be involved in the metastatization process. Among these, there is the homing of tumor cells into the bone correlated to several adhesion molecules, such as CD44 [3]. Once reached the bone microenvironment, other factors (including receptor activator of nuclear factor kappa-B ligand-RANKL, TGFß and parathyroid hormone-related protein-PTHrP) play a role in increasing matrix resorption and inhibiting osteoblastogenesis process, creating a “vicious cycle” of bone destruction [4].

Considering this background, it is easily understandable the importance to set up a personalized bone targeting therapy, to prevent the ineluctable reduced quality of life and worse survival outcomes due to skeletal complications [5]. Currently available bone-targeted agents (BTAs) are mainly represented by bisphosphonates and denosumab, both potent inhibitors of matrix resorption working through different mechanisms. They should be started once bone metastases are identified with the main aim of delaying or preventing SREs (consisting in pathological fractures, radiotherapy and surgery to bone, spinal cord compression and hypercalcemia) [6].

Bisphosphonates, analogues of pyrophosphate, are concentrated in active bone remodeling sites and can induce osteoclast death by endocytosis [2]. Nitrogen-containing bisphosphonates, such as zoledronic acid (ZA), are small molecules that hook in hydroxyapatite binding sites placed on bone mineral surfaces. When osteoclasts start to digest bisphosphonate-steeped bone matrix, the drug is released into their cytoplasm; once inside the cells, it binds to farnesyl pyrophosphate synthase, interfering with mevalonic acid pathway, leading to apoptosis [7]. More recent studies have shown that bisphosphonates, particularly ZA, can get involved in angiogenesis pathway, blocking the process of combination of angiogenesis and osteogenesis by inhibiting osteoclastogenesis, via platelet derived growth factor (PDGF) signaling [8]. In CRPC setting, ZA is the only nitrogen-containing bisphosphonate able to reduce SRE and bone pain [9]. Although it is considered cost effective and generally well tolerated, ZA is a drug administered only intravenously and could impair renal function. Conversely, denosumab is a human IgG2 recombinant monoclonal antibody, which targets and binds to RANKL. This abovementioned link prevents the activation of its receptor, known as RANK, placed on the surface of osteoclasts and osteoclasts’ precursors. Inhibition of the RANKL–RANK interaction blocks osteoclast formation, function and survival, decreasing bone resorption [10,11]. Differently from ZA, denosumab is administered subcutaneously and its clearance do not depend on kidney function. When compared to ZA, denosumab resulted to be better in terms of prevention of time to first SRE [12]. Both ZA and denosumab have been approved in castration-resistant PCa with skeletal involvement; conversely, patients with metastatic hormone-sensitive disease do not benefit from the addition of BTAs to standard of care active treatment [13].

Due to their potent inhibition of bone resorption, bisphosphonates and denosumab share an important adverse event: ONJ. This entity has been defined as the presence of exposed bone in the maxillofacial region that does not heal within 8 weeks after identification, in a patient with exposure to an antiresorptive agent without an history of radiation therapy to the same region [14]. A considering number of manuscripts have characterized a sub-group of patients, up to a quarter according to some authors, that could have different clinical presentations. In fact, it has been described a particular subtype of osteonecrosis of the jaw, known as “non-exposed MRONJ”, characterized by the absence of exposed bone on clinical inspection. In these cases, the clinical manifestations can range from dentally unexplained pain, presence of an intraoral fistula, mandibular fracture or swelling. Another aspect of the definition has been identified in the temporal criterion of 8-week bone exposure/probing of bone, which may not be applicable to all cases, thus causing diagnosis and treatment delay [15]. The pathophysiology of ONJ is not completely clear, but favoring factors are genetic predisposition, infections, bone vascularization and turnover [16]. In particular, periodontal and periapical infection could increase ONJ risk by altering osteoclast function and quantity. The composition of oral microbiota has been documented as a contributive factor in the host response to ONJ, altering systemic leukocyte’s function and TNF-alpha and RANK gene expression. Some systemic diseases, such as diabetes and rheumatoid arthritis, should be mentioned considering their role in promoting inflammation and inducing changes in immune cell function. When analyzing the relationship of ONJ with angiogenesis, it must be underlined the role of ZA, that is implicated in the inhibition of vascular endothelial cell proliferation and migration, in the regulation of mesenchymal stem cell differentiation and in the reduction of osteopontin levels in both maxilla and mandible. Another suggested mechanism is an altered gingival fibroblast function in soft tissues [17]. ONJ has been related not only to the exposure to BTAs, but also antiangiogenic drugs, such as bevacizumab [18], tyrosine kinase inhibitors (i.e., sunitinib, cabozantinib) mammalian target of rapamycin inhibitors (everolimus, temsirolimus), radiopharmaceuticals (i.e., radium223), selective estrogen receptor modulators (i.e., raloxifene), immunosuppressants (i.e., methotrexate, corticosteroids), and monoclonal antibodies (i.e., infliximab, adalimumab) [19]. Furthermore, recent evidence suggests a possible correlation of tocilizumab, a humanized anti-interleukin-6-receptor monoclonal antibody targeting interleukin-6 (IL-6) pathway, with ONJ. This monoclonal antibody, known for its use in rheumatoid arthritis, has recently been administered experimentally in patients affected by Sars-Cov-2-related pneumonia [20].

BTAs are pivotal treatments in patients with metastatic CRPC with bone involvement to combine to active oncologic therapies with the aim to prevent or delay SREs and also improve OS [21,22], so there is a relevant clinical need to properly prevent, address and manage possible toxicities related to BTAs. With regards to ONJ, this is a quite fearsome and potentially disabling side effect of these agents that needs to be adequately prevented and promptly recognized and treated to avoid chronic complications.

The aim of this review of the literature is to report the value of BTAs in PCa and their toxicities with a specific focus on ONJ, underlying the possible differences between ZA and denosumab.

## 2. Bone Targeting Agents in Hormone Sensitive and Castration Resistant Prostate Cancer

Bone health is becoming a pivotal therapeutic goal throughout the entire PCa natural history. There is a consensus on the need to include BTAs as a part of the treatment algorithm of PCa, but there is still a debate on the optimal starting timing and the most appropriate antiresorptive drug.

The need to properly address bone health preservation in the treatment approach to PCa is underlined by the inclusion of specific bone-related efficacy endpoints in clinical trials of novel androgen receptor-targeted agents (ARTA) (i.e., metastasis-free survival, time to first skeletal-related event) [23,24,25]. It has to be kept in mind that the backbone of PCa treatment is ADT, that is the main predisposing cause of bone fragility. The implicated mechanisms are mainly two: induction of sarcopenia, which increases the percentage of fat body mass, and increased bone turnover, that induces rapid and non-reversible microarchitectural damage and slow body mass loss. These quantitative and qualitative bone alterations result in an elevated risk of pathological and non-pathological fractures, typically occurring during the first year of therapy, because of rapid bone turnover [26]. So, skeletal involvement in PCa can be caused by two mechanisms: drug related, creating the so-called “cancer treatment-induced bone loss” (CTIBL), and disease related, due to the presence of metastases, causing complications known as SREs.

At the current moment, none of the bisphosphonates are approved for CTBIL treatment in metastatic HSPC since no benefit in OS has been shown in several phase III trials [27,28,29]. Denosumab (administered every 6 months) can be prescribed for ADT-induced bone loss prevention in men with increased fracture risk. In fact, the HALT trial showed a reduction in new vertebral fractures’ incidence and increased bone mineral density compared with baseline at all measured skeletal sites, including lumbar spine, hip and radius [30]. 

BTAs have been studied in both hormone sensitive and castration resistant setting.

A combined analysis of two randomized studies from 2003 investigated the use of infusive pamidronic acid 90 mg versus placebo every 3 weeks for 27 weeks in advanced PCa with skeletal metastases progressed after hormonal therapy [31]. The efficacy of pamidronic acid, evaluated with SRE proportion, pain reduction and use of analgesic was not superior to the control arm.

Another trial analyzed the use of oral clodronate acid 2080 mg daily versus placebo for up to three years in hormone-sensitive metastatic PCa [32]. The primary endpoint was symptomatic bone progression-free survival that resulted improved in the experimental arm (hazard ratio-HR = 0.79, 95% confidence interval-CI = 0.61–1.02; *p* = 0.066). The secondary endpoint of OS also favored the experimental arm (HR = 0.80, 95% CI = 0.62–1.03; *p* = 0.082). Adverse events (AEs) were almost comparable in the two arms, with only increased lactate dehydrogenase and hypocalcemia more frequent in the clodronate acid arm.

Numerous studies explored the efficacy of ZA in advanced PCa setting. The first phase III trial evaluated intravenous ZA at 4 mg and at 8 mg versus placebo every 3 weeks for 15 months in patients with hormone-refractory PCa with bone involvement [33]. After an amendment, patients in treatment with 8 mg ZA were switched to 4 mg dosage, due to higher rate of creatinine increase. The incidence of SRE, the primary endpoint, favored both experimental arms versus placebo with 33.2% versus 44.2% (difference versus placebo = −11.0%, 95% CI = −20.3% to −1.8%; *p* = 0.021) in 4 mg arm and 38.5% (difference versus placebo = −5.8%, 95% CI = −15.1% to 3.6%; *p* = 0.222) in the 8/4 mg arm. The safety analysis showed increased hypocalcemia, hemoglobin decrease and renal impairment in the experimental arms; however, the incidence of ONJ is not reported.

The phase 3 Cancer and Leukaemia Group B (CALGB) 90202 trial investigated the use of ZA in the earlier phase of hormone-sensitive prostate cancer with bone metastases. Patients were randomized between ZA at 4 mg or placebo every 4 weeks, with continuation with ZA in case of progression to castration-resistant PCa [34]. Unfortunately, the primary endpoint of SRE was not met. In fact, median time to SRE was 31.9 (95% CI 24.2–40.3) in the active arm and 28.8 months (95% CI 25.3–37.2) in placebo arm (HR 0.97, 95% CI 0–1.174; *p* = 0.385).

An important aspect that has been investigated is the potential role of BTAs in delaying disease progression from hormone-sensitive to castration resistant state. The ZAPCA trial compared ZA combined to ADT versus ADT alone in 224 patients with newly diagnosed metastatic PCa with bone metastases [35]. Primary objectives where time to treatment failure (TTF), time to SRE and OS. The combination therapy failed to show statistically significant improvement in TTF, with 12.4 months versus 9.7 months in experimental and control arm respectively (HR 0.75, 95 % CI 0.57–1.00; log rank *p* = 0.051). However, in a subgroup analysis of patients with baseline PSA values < 200 ng/mL, TTF significantly favored the ZA group with 23.7 months versus 9.8 months in the control group (HR 0.58, 95 % CI 0.35–0.93; log rank *p* = 0.023). Also time to SRE was improved in the combination arm with 64.7 months versus 45.9 months in the control arm (HR 0.58, 95 % CI 0.38–0.88; log rank *p* = 0.009).

Furthermore, the randomized controlled multiarm multistage STAMPEDE trial showed no improvement in OS in patients with HSPC with and without metastases receiving ADT (plus optional radiotherapy) and ZA or ZA alone compared with docetaxel and standard of care with or without ZA [29].

Several preclinical data suggested that the use of BTAs may prevent development of bone metastases in patients with CRPC [36]. The ZEUS trial investigated the role of ZA (every 3 months for ≤4 years) combined to standard PCa treatment for the prevention of bone metastases in high-risk nonmetastatic PCa. ZA added to standard therapy compared to standard therapy alone showed no significant improvement in bone metastasis-free survival at 4 years [28]. On the other hand, in a similar cohort of men, denosumab delayed time to first bone metastasis and increased metastasis-free survival, compared with placebo: a large phase III randomized trial explored denosumab in non-metastatic CRPC with high risk of developing bone metastasis [37]. High risk was based on PSA levels ≥ 8.0 μg/L or PSA doubling time ≤ 10.0 months, or both. A total of 1432 patients were randomized to receiving intravenous denosumab 120 mg every 4 weeks or placebo. Primary endpoint was bone-metastasis free survival, namely the first occurrence of bone metastasis or death. Denosumab improved significantly bone-metastasis free survival with a median 29.5 months versus 25.2 months (HR 0.85, 95% CI 0.73–0·98, *p* = 0.028). Exploratory endpoints of PFS and OS did not differ between the two groups.

Both ZA and denosumab are indicated for SRE prevention in patients with CRPC at the first radiological evidence of skeletal metastases. In a benchmark double-blinded randomized phase III study, 1904 men with metastatic CRPC where randomized to receive denosumab 120 mg or ZA 4 mg every 4 weeks [12]. Primary endpoint was time to SRE, evaluated as non-inferiority. Results were positive for the experimental arm, with median time to SRE of 20.7 months for denosumab and 17.1 months for ZA (HR 0.82, 95% CI 0.71–0.95; *p* = 0.0002 for non-inferiority; *p* = 0.008 for superiority). Exploratory analysis of OS and progression-free survival (PFS) did not show a statistically significant difference between the two arms. Safety was generally consistent with previous studies, with most frequent AEs being back pain, nausea, decreased appetite and fatigue. Hypocalcemia occurred more frequently with denosumab (121 patients, 13%) than with ZA (55 patients, 6%), (*p* < 0.0001). Further analysis highlighted that denosumab decreases the risk of symptomatic skeletal events (SSE) compared with ZA [38]. Of note, OS is not a primary endpoint in any of these studies, but it is included among secondary endpoint or post hoc analyses. In the study by Francini et al. [21], BTAs were associated with a benefit in OS in 745 patients with CRPC with bone metastasis receiving first line abiraterone acetate with prednisone. Furthermore, a post-hoc analysis of the COU-AA-302 study showed a survival benefit of the combination of BTAs with abiraterone acetate with prednisone compared with BTAs and prednisone alone (HR 0.71, 95% CI 0.50–1.00; *p* = 0.05) [39].

The results of the trials discussed in this section are reported in Table 1.

In addition to the abovementioned drugs, another class of therapies directed to bone metastases are radiometabolic treatments, like radium-223. The ALSYMPCA trial showed a significant OS benefit and prolonged time to first SSE of radium-223, a calcium mimetic alpha-emitting agent, compared with placebo [40]. As a matter of fact, different results emerged from the ERA-223 [41] and the EORTC 1333/PEACE [42] phase III studies. In these trials, the new radiopharmaceutical’s role has been debunked; in fact, to limit the risk of pathological fractures, radium-223 should not be administered together with classical BTAs and abiraterone plus prednisone or enzalutamide [42,43].

According to the latest guidelines on skeletal metastases management, ZA should be administered endovenously at the dose of 4 mg every 4 weeks even if 12-weekly schedule does not seem to be inferior to standard administration in terms of reducing the risk of skeletal events [43,44,45]. On the other hand, denosumab should be given subcutaneously every 28 days at the dose of 120 mg. As for ZA, the 12-weekly schedule is an alternative treatment strategy [45]. In addition, this longer-interval dosing will be also verified in the ongoing trial (REDUSE-NCT02051218). It is considered a proper clinical practice to co-administer a daily implementation of calcium and D vitamin and to suggest regular physical activity to prevent bone density loss. The duration of BTAs therapy currently recommended is at least two years; nevertheless, is widely accepted to prolong the period, assessing tolerability, clinical conditions and risk of skeletal events [2,13].

## 3. Adverse Events of Bone Target Agents

Although bisphosphonates and denosumab are generally well tolerated, it is important to prevent and early recognize any AEs that could occur. The rate and severity of AEs are similar between this two types of BTAs, despite having different mechanisms of action [46,47,48].

### 3.1. Toxicity and Safety Profile of Bisphosphonates

The toxicity profile of bisphosphonates may differ according with the modality of administration–orally or intravenously. Upper gastrointestinal tract adverse events due to mucosal irritation, such as nausea, vomiting, epigastric pain, esophagitis and dyspepsia, are common with the oral formulation, used mainly to treat post-menopausal osteoporosis [49]. In order to reduce the risk of this side effect, it is recommended to take the daily dose of the drug (alendronate) early in the morning, remaining in orthostatic position for at least 30 min to promote a rapid gastrointestinal transit of the tablet. In case of esophageal symptoms or sever gastrointestinal disorders, it is necessary to discontinue the treatment. Conversely, when bisphosphonates are used intravenously the most frequent early AE is a characteristic systemic reaction, known as “acute phase response”, consisting in bone pain, post-infusion pyrexia, fatigue, arthralgia, myalgia, stiffness and arthritis with subsequent joint swelling [50]. These symptoms are generally solved in a spontaneous way within a few days and can be managed with antipyretics.

Particular attention should be paid to serious adverse reactions, which can occur even after a long time, including renal toxicities, hypocalcemia and secondary hyperparathyroidism, osteonecrosis of the jaw (ONJ) and more rarely ocular events, atrial fibrillation and atypical features of the femoral diaphysis [49,50].

Nephrotoxicity, consisting of acute kidney injury (AKI) or glomerular disease, could be related to the highly affinity of bisphosphonates for some organism ions, including calcium, and their ability to create complexes which can be deposited in the kidney, even though the exact mechanism of this toxicity is still unknown. It may also be related to cumulative dose of the drug [49,50,51,52,53,54]. In order to prevent and/or reduce kidney damage, renal function should be monitored periodically, before and during treatment, by evaluating creatinine clearance (CrCl) and adjusting doses in patients with partially compromised renal function (CrCl between 30–60 mL/min). The administration of bisphosphonates should be avoided in case of severe renal failure (CrCl < 30 mL/min) [49,54,55].

Hypocalcemia, defined as a total serum calcium levels <8.8 mg/dL (<2.1 mmol/L) in the presence of normal plasma protein concentrations, is a typically intravenous bisphosphonates effect, uncommon with oral administration, caused by the potent inhibition of osteoclast-mediated bone resorption by these kinds of compounds. Along with the decrease of serum calcium, also the levels of serum phosphorus drop, with the secondary rise of parathyroid hormone (PTH) [50,56]. Hypocalcemia may be completely asymptomatic or produce severe signs and symptoms such as muscle spasm, paresthesia, changes in mood, hypotension and fatigue, up to more severe conditions as laryngospasm, seizures, arrhythmias and lethargy [50,57]. Subjects affected by hypoparathyroidism, parathyroid dysfunction, vitamin D deficiency, hypomagnesaemia and with impaired renal function are more susceptible to develop this AE [49,58,59,60]. An appropriate orally supplementation of vitamin D and calcium is required to avoid or reduce bisphosphonates hypocalcemia.

ONJ is described by the task force of the American Society for Bone and Mineral Research as the bone exposition in the maxilla-facial area that not recover within eight weeks after the identification by health care provider [61]. In patients treated with intravenous bisphosphonates for bone metastasis the incidence is estimated to range between 1 in 10,000 and 1 in 100,000 patient-treatment years and the risk is directly proportional to the duration of treatment [61]. ONJ will be described extensively in a dedicated section of this review.

### 3.2. Toxicity and Safety Profile of Denosumab

The safety profile of denosumab is similar to bisphosphonates, although it inhibits osteoclastic bone activity through a different pathway and it is administered subcutaneously. Neuromuscular and skeletal events, nausea, anemia, fatigue, atypical bone fractures, hypersensitivity, hypocalcemia, hypophosphatemia, ONJ and dermatologic reactions are the most frequent reported AEs. Electrolyte disorders and ONJ require more attention [46,62,63]. It is recommended to start an oral administration of vitamin D and calcium to treat or prevent hypocalcemia and to monitor periodically serum creatinine, vitamin D, calcium, phosphorus and magnesium. Patient should also pay attention to maintaining a good oral hygiene and regular dental check-ups. In case of renal impairment dosing adjustments are not required [62].

In a recent interesting meta-analysis, Chen et al. demonstrated an increased occurrence of hypocalcemia and ONJ with denosumab, compared to zometa [64]. On the other hand, a significantly lower incidence of renal adverse events and acute phase reactions was observed in patient treated with denosumab [46]. This makes denosumab the BTA of choice in case of renal failure and at the same time it is contraindicated in case of pre-existing hypocalcemia. Moreover, the same work evidenced the superiority of denosumab in delaying time to pain severity in comparison with zometa, also reducing the use of analgesic drugs [46].

Adverse events of BTAs are reported in Table 2.

## 4. Osteonecrosis of the Jaw: Pathogenesis, Clinical Presentation, Diagnosis and Treatment

The pathophysiology of ONJ caused by BTAs is still not fully understood. Several factors are thought to be involved in the development of this condition, especially due to the unique location of the disease [64].

One of the leading causal hypothesis is the suppression of bone remodeling and turnover due to the inhibition of osteoclasts activity and the accumulation of nonviable osteocytes [65]. Infections and inflammation are other frequent findings in ONJ. Reduced bone turnover is likely to diminish the host response against external pathogens. Moreover, pre-existing bacteria and biofilm, mainly composed of actionomyces bacteria class, have been found in mucosa and teeth near the necrosis area, which suggests that a chronic underlying inflammatory process like periodontal disease could favor ONJ [66,67]. The role of macrophages and monocytes is also likely involved. Inhibited similarly to osteoclasts, these cells of the immune system are less responsive to local infectious agents [68].

Avascular necrosis is another possible cause contributing to ONJ. Angiogenesis inhibition is caused mainly by the decrease circulating levels of vascular endothelial growth factor (VEGF) caused by bisphosphonates. Interestingly, cases of ONJ have been reported in patients treated with VEGF inhibitors. However, angiogenesis inhibition has not been reported with denosumab [69].

The specific anatomical localization of ONJ has also been linked to the continuous micro traumas of the lower jaw caused by mastication [70].

Interestingly, the rate of ONJ seems to be higher in cancer patients compared to non-oncologic patients, especially due to the higher doses and longer period of treatment with bisphosphonate or denosumab [71]. Intravenous bisphosphonates, and particularly zolendronic acid, have higher rate of ONJ compared to oral treatments, like pamidronate [61]. Moreover, a study on Guinea pigs, showed that a ‘’drug holiday’’ in the infusion of ZA with prophylactic antibiotics reduces the frequency of ONJ. However, the effect of paused schedule of ZA in humans in terms of reduction of ONJ rate has not been investigated [72].

Concomitant therapies have also an important role in increasing ONJ risk. Anticancer agents like VEGF and VEGF receptor inhibitors, cytotoxic chemotherapy (e.g., anthracyclines), and mammalian target of rapamycin (mTOR) inhibitors have all been reported to an increased risk of ONJ [73].

Local surgery procedures and local pre-existing oral and teeth diseases like periodontal disease, periapical abscesses, dental caries and bone exostosis also increment the rate of ONJ [74,75].

Clinical presentation of ONJ may vary from an asymptomatic area of necrotic bone which remain undetected for as long as years to a painful bone enlargement with impaired jaw mobility and soft tissue ulceration, swelling and edema [76]. Another peculiar symptom that may appear is the numb-chin syndrome (NCS), characterized by sensorial neuropathy of the inferior alveolar nerve. This clinical manifestation could be the first sign of an underlying ONJ [77]. Around two third of ONJ arise in the mandible compared to one third in the maxilla [61]. Radiological confirmation is based primarily on orthopantomography or computed tomography (CT) scan. While radiological findings are often aspecific, the most frequent findings are areas of altered mineralization, focal sclerosis and thickened lamina dura [78].

Treatment of ONJ lacks data from solid prospective trials. Conservative management is currently preferred compared to aggressive surgical management [79]. Conservative measures include limited surgical intervention with debridement and removal of necrotic tissue, oral hygiene comprehensive of antibiotic rinses, systemic antibiotics and pain control drugs [80]. More extensive surgical approaches could be reserved for refractory ONJ non-responsive to conservative management [81]. It is not clear if the suspension of bisphosphonate agents and denosumab could benefit the resolution of ONJ even if some data suggests a more rapid improvement [82]. Teriparatide is a recombinant parathyroid hormone, evaluated in a randomized controlled trial of 34 cancer patients with ONJ. The addition of teriparatide 20 mcg for eight weeks to vitamin D, calcium and standard of care measures, showed increased rate of recovery compared to placebo, with 45.4% versus 33.3% lesions resolved by 52 weeks [83]. However, the use of teriparatide in cancer patients remains limited due to concern for the anabolic and proliferative effect of the agent, even if no clear carcinogenetic effect has been demonstrated [84]. Another promising approach in the management of ONJ is the use of platelet-rich fibrin (PRF). Topical application of PRF on the necrotic bone, after surgical treatment, has the biological rationale of adding growth factor to the morbid tissue to promote bone and soft tissue recovery. Case series of patients with ONJ treated with PRF have reported favorable outcomes with reduced pain and faster healing time. However, its role in oncological patients it is still not established [85,86].

## 5. Osteonecrosis of the Jaw in Clinical Trials of Biphosphonates or Denosumab in Prostate Cancer Trials

Several phase III trials investigated the use of bisphosphonates or denosumab in various settings of PCa, from castration sensitive to castration resistant, and in patients with or without bone metastasis. However, in the safety analysis, especially in the older studies, the incidence and the severity of ONJ is not always mentioned or is reported differently, thus making an overall evaluation and comparison of this AE in the two different classes of agents difficult.

In the abovementioned studies by Small et al. [31], Dearnaley et al. [32], and Saad et al. [33], the incidence of ONJ is not reported.

The CALGB 90202 study published by Smith et al. [34] is the first phase III trial that reported ONJ in the AEs. Ten patients in ZA group and six patients in the placebo group reported a Common Terminology Criteria for Adverse Events (CTCAE) v.5.0 Grade 3 ONJ, that correspond to a ONJ with severe symptoms that need elective intervention. Grade 1 and Grade 2 ONJ are not reported.

In the ZAPCA trial ONJ is reported in 2 patients (1.8%) of the ZA group while no cases of ONJ are reported in the placebo group [35]. It is not reported the CTCA Grade of ONJ.

In the phase III trial by Smith and colleagues [34] exploring denosumab in non-metastatic CRPC with high risk of developing bone metastasis, no patients in the control arm experienced ONJ, while 33 patients (5%) in active treatment had this adverse event. Further data on these 33 patients are reported. Risk factors for ONJ such as tooth extraction (*n* = 23, 70%), poor oral hygiene (*n* = 18, 55%), and dental appliance use (*n* = 16, 48%) were noted. As for the treatment, limited interventions were needed in 21 (64%) patients with this adverse event while two (6%) patients had to be treated with bone resection. The remaining ten (30%) patients where managed with conservative management such as oral therapies and antibiotics. As expected, also hypocalcemia was more frequent in denosumab arm, with 12 (2%) patients versus 1 (<1%).

In the phase III study conducted by Fizazi et al. [12] that randomized patients with metastatic CRPC to denosumab or ZA, ONJ occurred in 22 patients (2%) in the denosumab group and in 12 patients (1%) in the ZA group (*p* = 0.09). The majority of the 34 patients, 17 (77%) on denosumab and 10 (83%) on ZA, that experienced ONJ had a clinical history of tooth extraction, use of dental appliance or poor oral hygiene. Concurrent chemotherapy in patients with ONJ was recorded in 14 patients (64%) treated with denosumab and nine (75%) treated with ZA. Surgical intervention for ONJ had to be performed in 12 patients in the denosumab arm; of these, 10 patients (45%) had conservative surgery and 2 (9%) underwent bone resection. In the ZA arm, 3 patients (25%) had to be treated with conservative surgery while 1 (8%) underwent demolitive surgery. Resolution of ONJ, meaning complete mucosal reepithelization, was achieved in 4 patients (18%) in the denosumab group and 1 patient (8%) in the ZA group.

## 6. Conclusions

Bone targeting agents are a backbone treatment of prostate cancer patients with bone metastases, that are a frail population liable of multiple complications. Bisphosphonates and denosumab, through different mechanisms of action, accompanies the oncologic systemic treatment in the great majority of patients with advanced prostate cancer. Their efficacy is similar even though some differences between the two compounds have been evidenced in several phase III trials. Even toxicities appear to be mostly equivalent, with some mild differences in terms of hypocalcemia. Osteonecrosis of the jaw is one the most invalidating adverse events and, even though is very rare, it has to be adequately prevented and promptly treated. The different definition and incomplete report of the adverse events in the clinical trials do not allow to have a clear estimate of the difficulties that the clinician has to face and manage throughout the course of these treatments, that are carried out for long periods. A homogeneous report and clinical management of these toxicities could help to improve the adherence and quality of life of patients with bone metastases.

## Figures and Tables

**Table 1:** Efficacy of Bone Targeting Agents in Prostate cancer: mCRPC = metastatic castration resistant prostate cancer; ZA = zoledronic acid, SRE = skeletal related events; PA = pamidronic acid; mHSPC = metastatic hormone sensitive prostate cancer; TTTG = time to treatment failure.

Study (Authors, Year)	Design	Patients Enrolled	Setting	Agent(s)	Primary Endpoint(s)	Results
Saad et al., 2002 [33]	Phase III, randomized	643	mCRPC	(1) ZA 4 mg vs. placebo (2) ZA 8 mg (later reduced to 4 mg) vs. placebo	SRE	(1) 44.2% vs. 33.2%; (95% CI = −20.3–1.8%; *p* = 0.021 (2) 38.5% vs. 33.2%; 95% CI = −15.13.6%; *p* = 0.222)
Small et al., 2003 [31]	Combined Two Phase III, randomised	350	mCRPC	PA vs. placebo	SRE Pain score	Difference not statistically significant
Fizazi et al., 2011 [12]	Phase III, randomized	1901	mCRPC	Denosumab vs. ZA	Time to SRE	20.7 vs. 17.1 months (HR 0.82, 95% CI 0.71–0.95; *p* = 0.0002 for non-inferiority; *p* = 0.008 for superiority)
Smith et al., 2012 [37]	Phase III, randomized	1432	mCRPC	Denosumab vs. placebo	Bone-metastasis-free-survival	29·5 vs. 25·2 months (HR 0.85, 95% CI 0.73–0·9, *p* = 0.028)
Smith et al., 2014 [34]	Phase III, randomized	645	mHSPC	ZA vs. placebo	Time to SRE	31.9 vs. 29.8 months (HR = 0.97 95% CI, 0–1.17; *p* = 0.39)
Kamba et al., 2017 [35]	Phase III, randomized	227	mHSPC	ZA vs. placebo	TTTF	12.4 vs. 9.7 months ((HR 0.75; 95% CI 0.57–1.00; *p* = 0.051)

**Table 2:** Adverse events of Bone Targeting Agents in Prostate Cancer. ZA = zoledronic acid, PA = pamidronic acid, ONJ = osteonecrosis of the jaw.

Study (Authors, Year)	Patients Enrolled	Agent(s)	ONJ	Hypocalcemia	Bone Pain	Constipation
Saad et al., 2002 [33]	643	ZA (4 mg and 8 mg) vs. placebo	n.a.	4 vs. 0 (G3-G4)	108 vs. 127	72 vs. 72
Small et al., 2003 [31]	350	PA vs. placebo	n.a.	n.a.	77 vs. 75	39 vs. 40
Fizazi et al., 2011 [12]	1901	Denosumab vs. ZA	22 vs. 12	121 vs. 55 (48 vs. 12 G3-4)	304 vs. 287 (back pain)	236 vs. 251
Smith et al., 2012 [37]	1432	Denosumab vs. placebo	33 vs. 0	12 vs. 2 (9 vs. 0 G3-4)	168 vs. 156	127 vs. 119
Smith et al., 2014 [34]	645	ZA vs. placebo	10 vs. 6	45 vs. 54 (7 vs. 3 G3-4)	96 vs. 60	n.a.
Kamba et al., 2017 [35]	227	ZA vs. placebo	2 vs. 0	n.a.	n.a.	n.a.
